# Prevalence of Various Traumatic Events Including Sexual Trauma in a Clinical Sample of Patients With an Eating Disorder

**DOI:** 10.3389/fpsyg.2021.687452

**Published:** 2021-08-19

**Authors:** Gry Kjaersdam Telléus, Marlene Briciet Lauritsen, Maria Rodrigo-Domingo

**Affiliations:** ^1^Psychiatry, Aalborg University Hospital, Aalborg, Denmark; ^2^Institute of Communication and Psychology, Psychology, Aalborg University, Aalborg, Denmark; ^3^Department of Clinical Medicine, Faculty of Medicine, Aalborg University, Aalborg, Denmark

**Keywords:** eating disorder, anorexia nerviosa, bulimia nervesa, trauma, sexual trauma

## Abstract

**Objective:** Eating disorder (ED) and trauma have often been associated, and there is evidence that early experiences of traumatic events are associated with subsequent ED. Research results point toward an increased prevalence of sexual trauma in individuals with ED, and it has been suggested that sexual trauma precedes and contributes to the development of ED. The aim of this study was to assess the prevalence of sexual trauma as well as other types of traumatic life events in a clinical sample of children, adolescents, and adults with ED.

**Method:** Patients (*N* = 329), median age 16.9 [Interquartile Range (IQR):4.5], diagnosed with various EDs in a specialized ED unit were included.

**Results:** The majority (67%) of patients with ED reported at least one traumatic life event at time of assessment such as bullying (32%), loss (24%), or accidents (11%). Nineteen per cent of the patients reported having been the victim of a sexual trauma or another sexual traumatic event distributed as follows in terms of severity: 13% had been the victim of a negative experience associated with sex; 57% reported having experienced sexual assault other than rape; and 30% had been the victim of severe forms of sexual assault. The median time between the sexual trauma and the ED diagnosis was 3.4 years (IQR: 6.6). The median time between the sexual trauma and the onset of ED symptoms was 0 years (IQR: 5). The study results imply that the sexual trauma could be experienced either prior to or after onset of ED symptoms.

**Conclusions:** Sixty-seven per cent of the patients with an ED reported traumatic life events at time of assessment, whereby 19% reporting negative sexual experiences or sexual abuse. However, sexual trauma does not necessarily play a causal role in the development of EDs.

## Introduction

Stressors are often believed to play a crucial role as risk factors in the onset of eating disorders (ED) (Smyth et al., 2008). Traumatic events (TEs) are one type of stressors associated with risk for an ED (Molendijk et al., 2017). Trauma are often categorized into various groups, including sexual abuse, physical abuse, and emotional abuse. TEs can include both single- and multiple incident(s) and may be highly invasive and/or interpersonal in nature. One example of severe TE could be multiple experiences of victimization in connection with extended childhood neglect disrupting the parent-child attachment system and/or abuse (Zlotnick et al., 1996; Ford and Kidd, 1998) including sexual trauma. Sexual trauma is defined as unwanted sexual contact and includes sexual touching, fondling, attempted rape, rape, and incest. In a systematic review, Chen et al. (2010) found a statistically significant association between sexual abuse and a lifetime diagnosis of anxiety disorder, depression, post-traumatic stress disorder, sleep disorders, suicide attempts as well as ED. The associations persisted regardless of the victim's sex or age when the abuse occurred (Chen et al., 2010). Likewise, studies including meta-analyses and reviews have concluded that a strong association between childhood maltreatment and ED exists (Smolak and Murnen, 2002; Stice, 2002; Madowitz et al., 2015; Caslini et al., 2016; Molendijk et al., 2017). The relationship between ED and trauma has been investigated in several studies. In a systematic review and meta-analysis focusing on the association between these distinct types of abuse during childhood and different ED subtypes, the authors concluded that while bulimia nervosa (BN) and binge eating disorder (BED) were associated with childhood abuse, anorexia nervosa (AN) showed mixed results (Caslini et al., 2016). In a recent study by Talmon and Widom (2021), it was found that childhood maltreatment was not a significant risk factor for AN or BN diagnoses or symptoms in adulthood. However, the study found that adults who retrospectively reported maltreatment, physical abuse, and sexual abuse reported significantly more symptoms of AN than those who did not. According to Brewerton (2007), childhood sexual abuse is a non-specific risk factor for ED. The spectrum of trauma linked to ED includes a variety of abuse and neglect. Trauma is more common in the bulimic ED subtypes compared to the non-bulimic ED subtypes and the findings linking EDs with trauma have been extended to children/adolescents and boys/men with EDs as well. Multiple episodes or forms of trauma are associated with EDs, and that trauma is not necessarily associated with greater severity of ED (Brewerton, 2007). However, only a few studies of TEs have been conducted in children and adolescents with ED, and they have primarily focused on females with an ED (Sanci et al., 2008; Jaite et al., 2012). Furthermore, most studies have focused on AN or BN, and therefore only a limited number of studies have included less specific categories of ED, including other specified feeding or eating disorders (OSFED), atypical AN, and atypical BN (Molendijk et al., 2017).

## Aim of the Study

The aim of this cross-sectional study was to analyze the prevalence at time of assessment of various types of traumatic events (TEs) including sexual TE (STEs) in a clinical sample of children, adolescents and adults with ED. Another aim was to search for potential differences between children/adolescents and adults with ED regarding the nature of STE. Finally, the aim was to investigate whether the reported STEs happened before or after disease onset.

## Method

### Study Population

**Figure 1:**
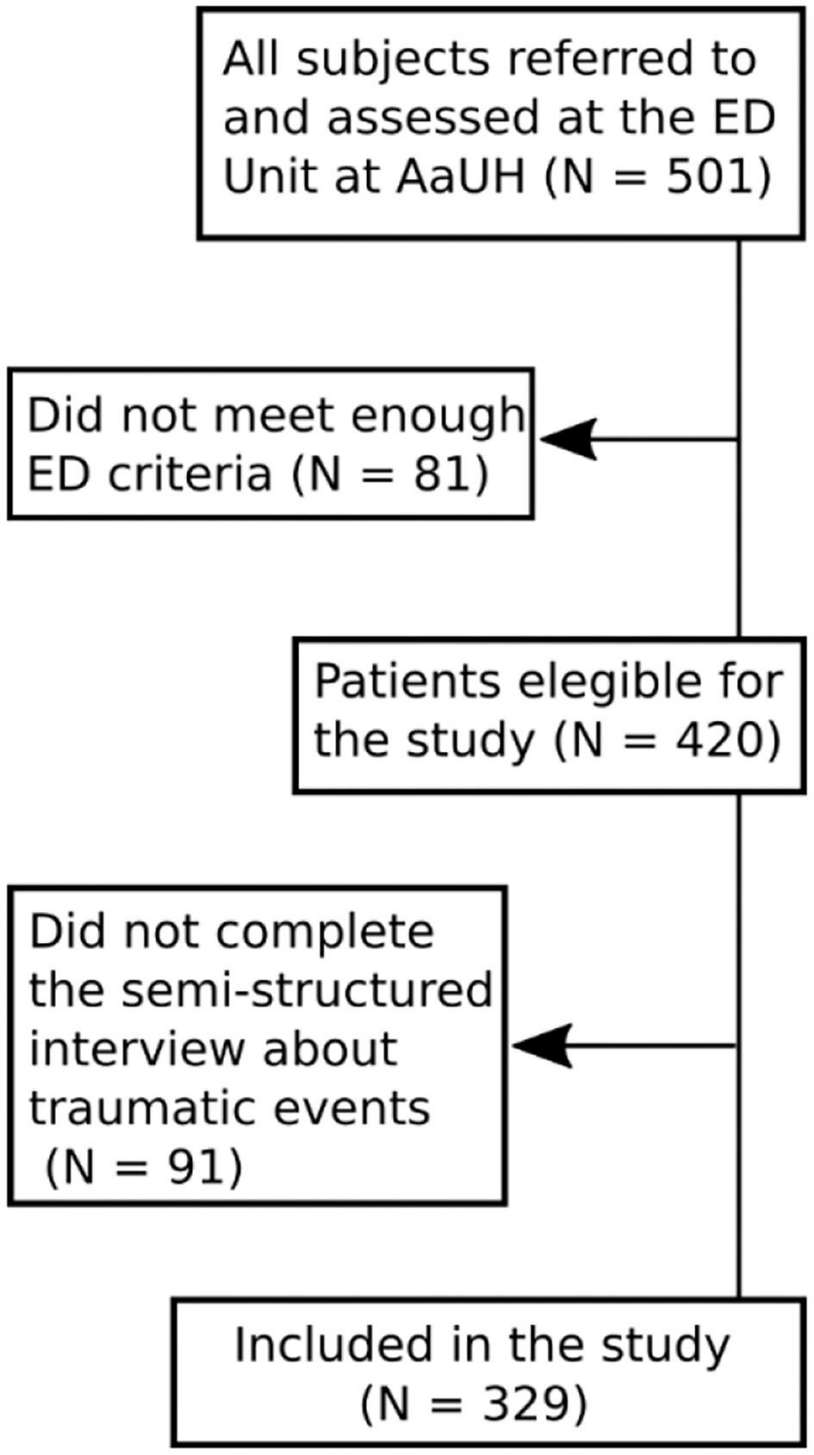
Flow chart showing study inclusion.

The study population consisted of all individuals referred to the Unit for Eating Disorders at Aalborg University Hospital, Denmark, between January 2009 and May 2014. The Unit for Eating Disorders is a specialized interdisciplinary ED unit providing in- as well as outpatient treatment for patients of all ages with an ED. The diagnostic categorization for this study was conducted retrospectively in accordance with DSM-5 criteria (American Psychiatric Association, 2013) based on the diagnostic interview conducted at the time of diagnosis. The assessment was carried out during the first hospital contact (repeated at re-referrals) at the ED unit. A total of 329 female and male patients, children, adolescents as well as adults, were included in this study. See flowchart in Figure 1.

### Diagnostic Assessment of ED

All assessment tools were administered by highly trained staff with extended experience in working with ED.

All patients were assessed for an ED according to a standardized diagnostic assessment battery that included the Eating Disorder Examination, edition 16.0D (EDE-16) (Cooper et al., 1989). Fully trained clinical psychologists administered the EDE-16 (Fairburn et al., 2008) interview after having received formal training before conducting the diagnostic interview. On-going co-rating and supervision were also provided. The EDE-16 assesses the frequency and severity of ED symptoms and behaviors indicative of an eating disorder over a 28-day period. The EDE-16 is scored on a 7-point scale (0-6). Cut-off for meeting a given ED criterion in this study was set at 3. EDE-16 has good internal consistency, discriminant and concurrent validity, and inter-rater reliability (Cooper et al., 1989; Fairburn et al., 1993; Wilfley et al., 2000).

The assessment likewise included information regarding the onset and developmental history of the ED.

### Assessment of Trauma

The assessment also included a semi-structured interview on various life experiences, including TEs. Thus, after conducting the EDE-16, the same clinical psychologist performed a semi-structured interview to obtain information on various life experiences, including crucial and/or traumatic life events such as sexual traumatic events, loss (including parental loss), bullying, or accidents. A text describing the nature, frequency and severity of the STE was also provided by the psychologist.

### Further Assessment

The ED assessment battery further entailed a medical examination including structured collection of certain clinical observations and patient-reported symptoms according to generally recognized medical complications in ED. A parental interview to collect anamnestic data was conducted as well, however these data were not included in this study.

### Definition of Variables

Age at assessment, which is also the time of inclusion in this study, was defined as age at time of diagnostic assessment with EDE-16. Patients were divided into two different age groups: children/adolescents when assessed before turning 18 and adults otherwise. Age at symptom onset for AN and atypical AN was defined as the onset of restrictive eating behavior and for BN and atypical BN as the onset of binge eating, purging and/or weight-controlling behavior. For AN binge-purge and other OSFED, onset of the first ED behavior among those mentioned above was chosen. BN behavior was determined as being present if the patient reported at least 12 episodes of BN binge and purging (or other compensatory behavior) in the 3 months prior to assessment. Compensatory behavior was a broad concept including vomiting, use of laxatives and/or diuretics, and extreme training (defined as exercise that exceeded several hours per day, caused distress if the individual was unable to exercise, exercise at inappropriate times and/or places, exercise that interfered with important activities, or exercise despite injury/illness/medical complication). The patient's BMI was calculated based on the height and weight measures obtained during the medical examination. If these measures were not available, patient-reported weight and height were used (collected with the EDE-16). As this study also includes children and adolescents, the WHO standards for weight-for-age and sex were used to compare the patient's BMI for diagnostic criteria. Duration of ED was defined as the difference between the patient's reported age at onset of ED symptoms (e.g., regulation in food intake, binge eating or purging behavior) and the date of the diagnostic interview.

Information from the interview was used to define the binary variables “bullying,” “accident,” “loss,” “sexual,” or “other,” each of which contain TEs experienced or not by each patient. The variable “bullying” refers to all forms of bullying experienced by the patient, including being bullied by peers, juniors, older youths or adults; “accident” refers to accidents experienced by the patient, including being the victim of an accident, being a witness closely involved in an accident, or being involved in the aftermath of an accident; “loss” includes parental loss (one or both parents) as well as loss of other family members such as step-parents, grandparents, siblings or children, and of close friends; “sexual” covers a wide range of events including any unwanted sexual contact such as sexual touching, fondling, attempted rape, rape, and incest. Finally, “other” covers any TE that could not be classified under the previous categories at the time of the interview. A patient was determined to have experienced a TE if any of the above TEs was present (variable “any type of TE”). The number of different TE types experienced by each patient was recorded in the variable “number of different TEs” and classified as “None” (no TE), “One” (a single type of TE), “Two” (two different types of events), or “Three or more” (at least three TEs in three different categories). The severity of the sexual trauma and sexual traumatic events was rated independently by two of the authors (GKT and MRD) based on the accompanying text from the interview. The rating was categorized as follows: (1) Negative experiences associated with sex; (2) Sexual assault other than rape; (3) Sexual assault including rape, repeated rape, and incest. The categorizations were conducted separately (blinded) and subsequently consensus rated.

### Statistical Analysis

All statistical analyses were performed in Stata14 (StataCorp, 2015). For categorical variables, the number of cases and percentages are reported, and for continuous variables the mean and standard deviation, except for highly skewed variables that are summarized using median, and first (Q1) and third (Q3) quartiles. No imputation of data was performed. Therefore, for variables where we do not have information on all patients, such as presence of lanugo, the percentage of lanugo is computed within the group of patients with available information. Chi-square tests for association or Fisher's exact tests were used to compare prevalence of TE between diagnostic groups or age groups. Continuous variables were compared between pairs of diagnostic categories using Wilcoxon rank-sum tests. The association between diagnostic category and number of different TE types was investigated using ordered logistic regression. For this analysis, the AN-R group was chosen as reference and odds ratios (OR) for the other groups compared to AN-R are reported. Results with p-values below 0.05 are considered statistically significant.

### Ethical Aspects

The study was evaluated by the North Denmark Region Committee on Health Research Ethics for ethical approval. Due to the nature of the study design, no further approval was needed. The study was approved by the local Data Protection Agency (the North Denmark Region) and conducted in accordance with the Helsinki Declaration. To preserve patient privacy in general and due to the sensitive nature of the study aim, it was decided to follow the regulations set by the Danish Data Protection Agency. Thus, caution was taken for groups consisting of fewer than four patients, and data are not reported in exact numbers but rather shown as informative percentages.

## Results

As reported, a total of 329 patients were included in the study (95.4% females and 4.6% males). Most of the included patients were children and adolescents with 225 (68%) patients in the group aged between 10 and 17 years and 104 (32%) patients in the age group 18 or above. Table 1 presents demographic and diagnostic characteristics of the study population divided by diagnostic categories. Median age at time of diagnosis was 16.9 years of age [15.0; 19.5] spanning between just over 11 years old and the early 30s, while median duration of illness at time of diagnosis was 1.7 years [1; 3.1], with some patients having had symptoms for a few months and others for over 20 years. In all diagnostic groups, over 90% of the patients were women. Patients with AN were younger than patients with BN (*p* < 0.001) at time of diagnosis and had had ED symptoms for a shorter duration of time (*p* < 0.001). BN and AN binge-purge were comparable, both in age at diagnosis and ED duration (*p* = 0.6 and 0.7, respectively), though BMI obviously differs. Compared to AN-R, patients with AN binge-purge were diagnosed at an older age (*p* < 0.001) and had been sick for a longer time (*p* < 0.001). Lanugo and peripheral cyanosis were mostly present in patients with AN restricting-type (AN-R) and AN binge-purge, but also in patients with other OSFED, whereas it was practically absent in patients with BN and atypical AN.

**Table 1:** Characteristics of the study population.

	**ED**	**AN-R**	**AN-BP**	**BN**	**OSFED**	**Other**
					**AN-atypical**	**OSFED**
Individuals	329	70 (21%)	42 (13%)	57 (17%)	69 (21%)	91 (28%)
**Sex** ^**a**^						
Female	314 (95%)	66 (94%)	> 95%	> 95%	> 95%	84 (92%)
Male	15 (5%)	4 (6%)	<5%	<5%	<5%	7 (8%)
Age at assessment^b^	16.9 [15.0; 19.5]	15.7 [14.4; 17.3]	18.0 [16.4; 21.9]	18.9 [16.4; 22.2]	16.8 [14.8; 19.4]	16.5 [15.0; 18.4]
Illness duration of ED^b^	1.7 [1.1; 3.3]	1.3 [0.9; 1.8]	3.1 [1.9; 5.5]	3.3 [1.7; 6.2]	1.6 [1.2; 2.8]	1.4 [1.0; 1.9]
BMI^c^	18.3 (3.6)	15.7 (1.5)	16.7 (1.3)	22.1 (3.0)	21.2 (3.5)	16.6 (2.0)
Binge eating^a^	84 (26%)	<5%	22 (52%)	57 (100%)	<5%	<5%
Purging behavior^a^	246 (75%)	45 (64%)	40 (95%)	57 (100%)	48 (70%)	56 (62%)
Lanugo^a^	61 (26%)	24 (43%)	13 (39%)	5%	<5%	20 (32%)
Peripheral cyanosis^a^	37 (15%)	18 (32%)	7 (21%)	0 (0%)	<5%	10 (16%)

*Overview of the diagnostic characteristics as well as patient characteristics of the study population. The number of patients in each specific diagnostic category as well as age at assessment (N = 329), sex (N = 329), duration of ED (N = 303), BMI (N = 318), and presence of binge eating (N = 329), purging behavior (N = 329), lanugo (N = 236), and peripheral cyanosis (N = 241) are presented. The percentages are computed based on the number of patients with non-missing information for each variable*.

a *N (%)*.

b *Years, median [Q1; Q3]*.

c *Mean (sd)*.

**Figure 2:**
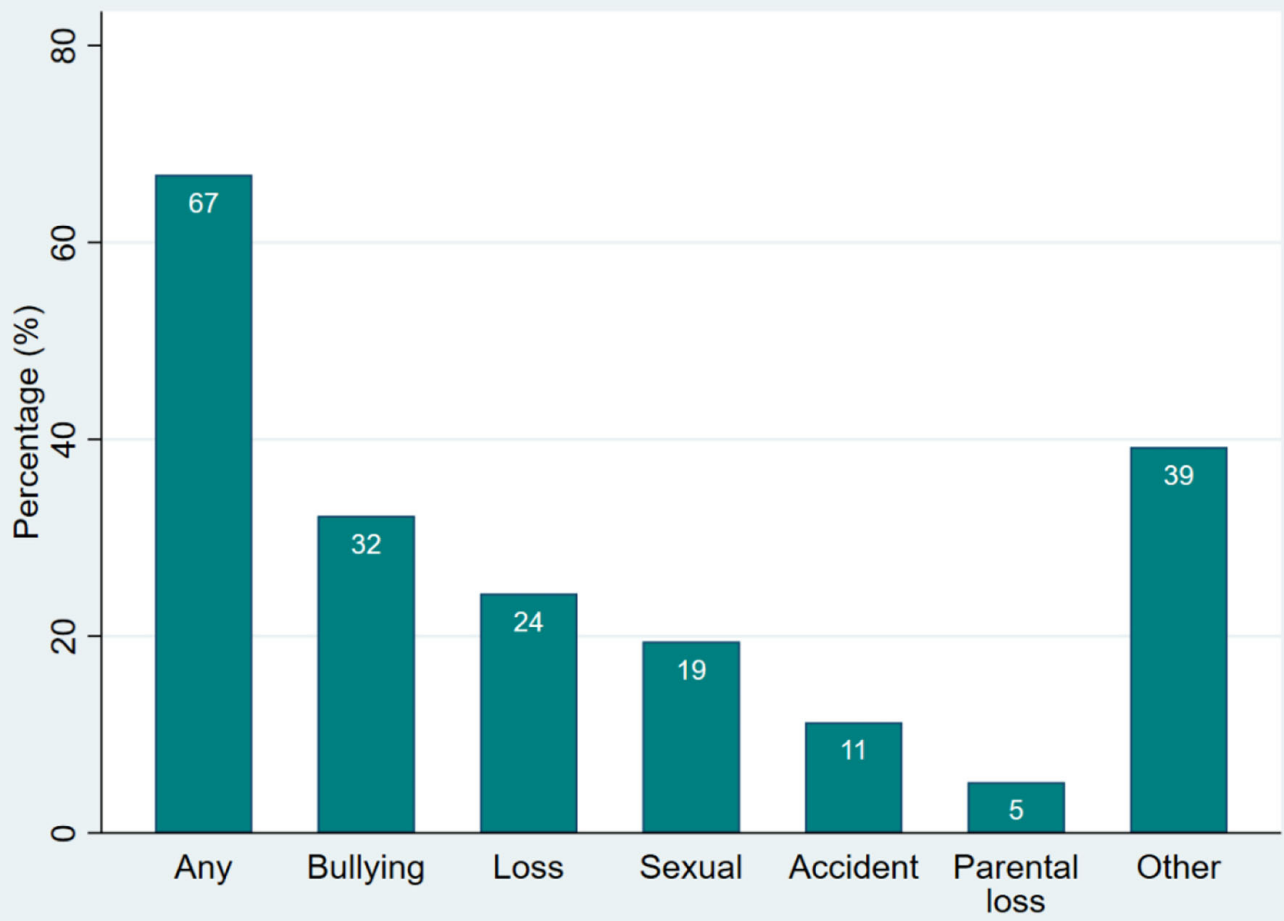
Types of TE reported by the patients. Percentage of patients (*N* = 329) with ED who have experienced each type of TE.

Sixty-seven per cent (95% CI [61%, 72%]) of the patients had experienced at least one TE (Figure 2), among which bullying was the most common (32%, 95% CI [27%, 38%]), followed by loss (24%, 95% CI [20%, 29%]) (including 5% (95% CI [3%, 8%]) parental loss), negative sexual experience/assault (19%, 95% CI [15%, 24%]), and accident(s) (11%, 95% CI [8%, 15%]). Thirty-nine per cent of the patients (95% CI [34%, 45%]) reported a TE that could not be included in any of these categories. The number and percentage of patients, divided by diagnostic ED categories, experiencing each type of TE are presented in Table 2. There were no statistically significant associations between diagnostic categories and the following variables: “Any TE,” “Bullying,” “Loss,” “Accidents,” “Parental loss,” or “Other TE.”

**Table 2:** Prevalence of different types of traumatic experiences by diagnostic category (number of patients and percentage per category).

	**AN-R**	**AN-BP**	**BN**	**OSFED**	**Other**	***P*-value**
	**(*N* = 70)**	**(*N* = 42)**	**(*N* = 57)**	**AN-atypical**	**OSFED**	
				**(*N* = 69)**	**(*N* = 91)**	
Any type	39 (56%)	30 (71%)	41 (72%)	47 (68%)	63 (69%)	0.3
Bullying	24 (34%)	13 (31%)	23 (40%)	20 (29%)	26 (29%)	0.6
Loss	10 (14%)	9 (21%)	19 (33%)	20 (29%)	22 (24%)	0.1
(Parental loss)	<4%	<4%	5 (9%)	3 (4%)	6 (7%)	0.5)
Sexual	10 (14%)	14 (33%)	14 (25%)	17 (25%)	9 (10%)	<0.01
Accident	6 (9%)	6 (14%)	5 (9%)	7 (10%)	13 (14%)	0.7
Other type of TE	18 (26%)	17 (40%)	25 (44%)	29 (42%)	40 (44%)	0.1

*The number and percentage of patients, divided by diagnostic categories, who have experienced the various types of TE. P-values from chi-square tests for association between presence of each*.

Table 3 presents the total number of different TE types experienced by each patient in the whole group and for each of the ED subtypes. It appeared that 109 patients (33.1%) had experienced no TE; 90 patients (27.4%) had experienced a single type of TE; 80 patients (24.3%) had experienced two different types of TEs; and 50 patients (15.2%) had experienced at least three different types of TEs. Nine (3%) patients reported experiencing more than three different types of TEs. The maximum reported number of different TEs is five, and no males reported more than three TEs. The association between the number of different TE types and the diagnostic category was statistically significant (*p* = 0.049). Specifically, the likelihood of having experienced more different TE types is significantly lower for the AN-R group than all other diagnostic subgroups except other OSFED. Indeed, the estimated OR compared to AN-R were highest for BN (OR = 2.4), followed by AN-BP (OR = 2.3), OSFED AN-atypical (OR = 2.0), and finally other OSFED (OR = 1.6) (95% CIs [1.3, 4.6], [1.1, 4.6], [1.1, 3.6], and [0.9, 2.8], respectively).

**Table 3:** Total number of different TE types experienced by each patient in the whole group and per diagnostic category.

	**ED**	**AN-R**	**AN-BP**	**BN**	**OSFED**	**Other**
	**(*N* = 329)**	**(*N* = 70)**	**(*N* = 42)**	**(*N* = 57)**	**AN-atypical**	**OSFED**
					**(*N* = 69)**	**(*N* = 91)**
None	109 (33%)	31 (44%)	12 (29%)	16 (28%)	22 (32%)	28 (31%)
One	90 (27%)	21 (30%)	8 (19%)	13 (23%)	17 (25%)	31 (34%)
Two	80 (24%)	11 (16%)	16 (38%)	16 (28%)	17 (25%)	20 (22%)
Three or more	50 (15%)	7 (10%)	6 (14%)	12 (21%)	13 (19%)	12 (13%)

*Number of different TE types experienced per patient divided by diagnostic category*.

Sixty-four females, corresponding to 19% (95% CI [15%, 24%]) of all patients, reported some type of STE while none of the 15 males reported STE. Among those reporting STE, the severity was distributed as follows: 13% of the patients reported a negative experience associated with sex; 57% reported having experienced a sexual assault other than rape; while 30% had experienced extreme forms of sexual assault including rape, repeated rape, and incest. The reported prevalence of sexual trauma was significantly associated with the diagnostic group (*p* < 0.01) with the lower prevalence in other OSFED (10%, 95% CI [5%, 18%]) and AN-R (14%, 95% CI [7%, 25%]) and somewhat higher in patients with AN binge-purge (33%, 95% CI [20%, 50%]), atypical AN (25%, 95% CI [15%, 36%]), and BN (25%, 95% CI [14%, 38%]), see Table 2.

**Figure 3:**
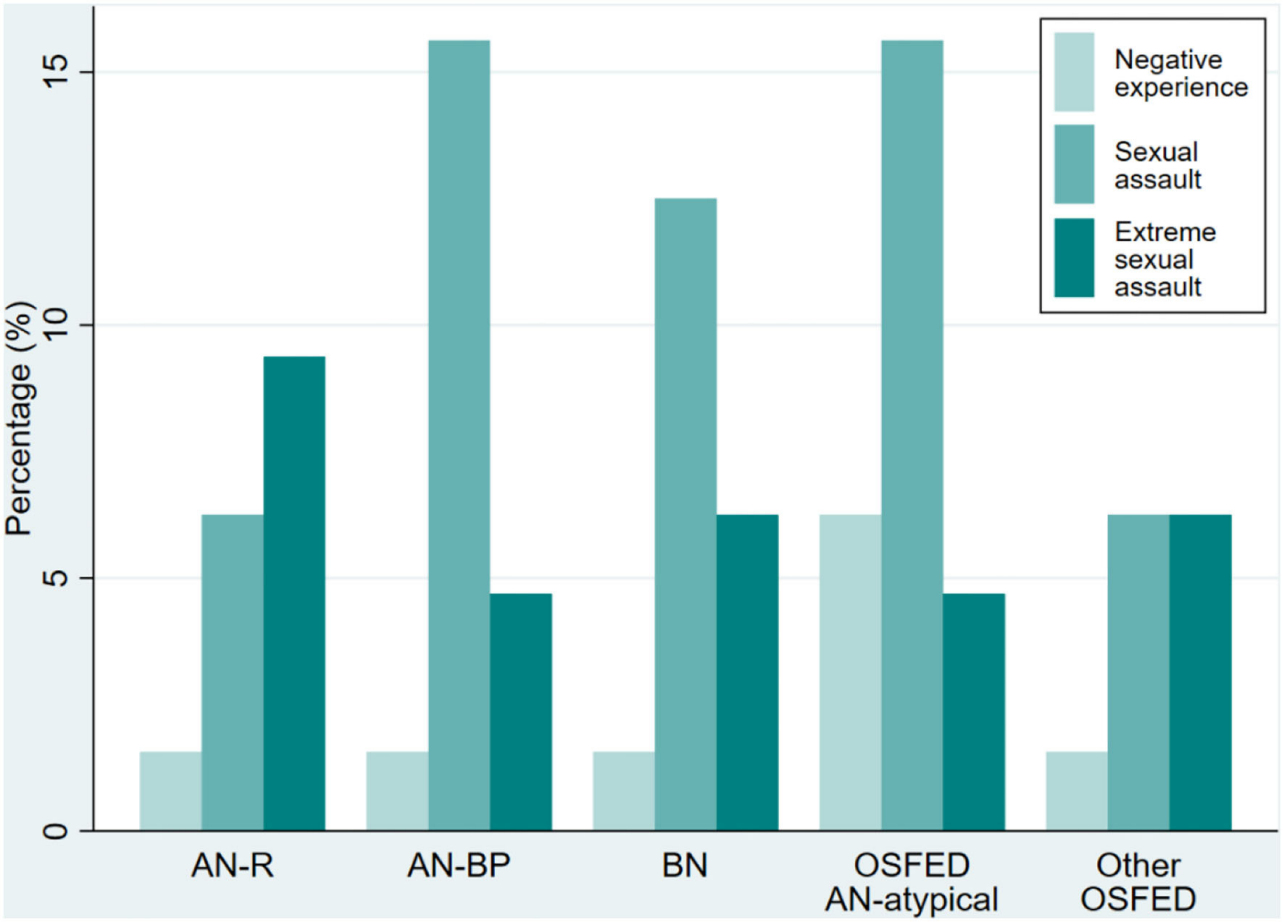
Severity of STE by diagnostic category. Distribution of the severity of STE for each diagnostic category (*N* = 329). Note that since far from all patients reported STE, the sum of the percentages in the bars for each diagnostic category does not add up to 100%.

Figure 3 displays the distribution of severity of STE by diagnostic category. Patients in the AN-R group experienced extreme sexual assault more often than any of the other ED groups, where sexual assault was the most commonly reported STE. Fifty-one of the patients suffering STE reported their age at the time the assault occurred. The median age at STE was 14 years [12; 16], with 6 (12%) patients aged 5 years or younger at the time of the STE and 7 (14%) having experienced the STE as adults.

**Figure 4:**
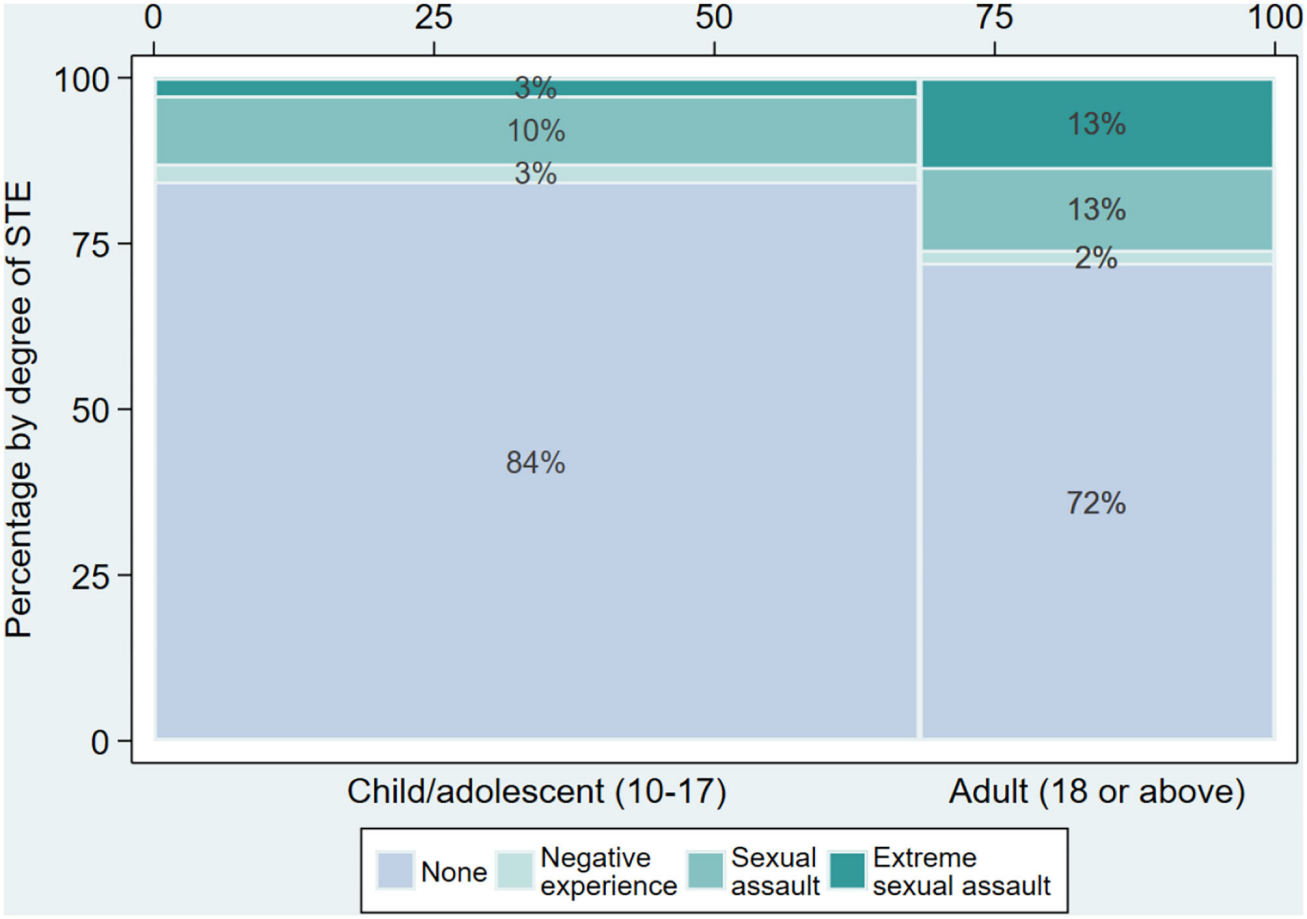
Severity of STE for children/adolescents and adults. This figure displays the distribution of presence and severity of STE divided by age group [*N* = 329, for children/adolescents (*N* = 225) and adults (*N* = 104), respectively]. The percentages are within each age group. The thickness of the bar for each age group reflects the size of the group (see percentage bar at the top).

Among the 64 patients that had experienced sexual traumatic events or sexual trauma, 35 (55%) were children/adolescents and 29 (45%) were 18 years or above. Figure 4 presents a more detailed description of the severity of STE divided by group (children/adolescents vs. adults). As shown, the reported STE in adults was 12 percentage points higher than in children (16 vs. 28%, chi-square test, p-value for association <0.001). A more profound difference between the two groups appeared when looking at the severity of STE; indeed, almost all of the adult-reported STEs were at least classified as sexual assault, and 48% were extreme sexual assault, compared to 17% among the STEs reported by the younger patients.

**Figure 5:**
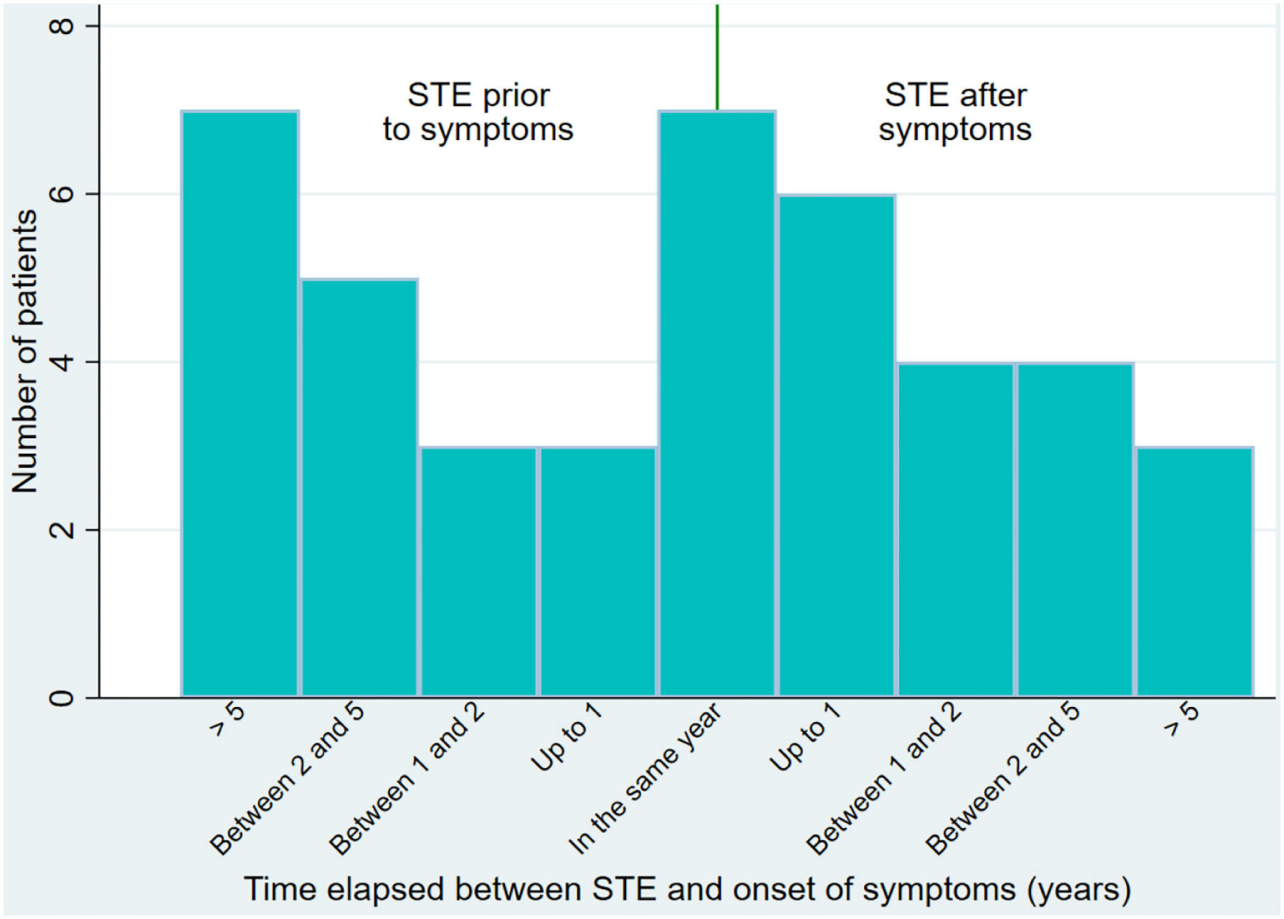
Time elapsed between STE and symptoms of ED. Bar plot of the time elapsed between the STE and the onset of ED symptoms for patients reporting both (*N* = 42).

The median time between the STE and the ED diagnosis was 3.5 years [1; 8], with 12 (23%) of the patients reporting less than a year between the sexual trauma and the ED diagnosis, and 10 (19%) of the patients reporting that the STE happened more than 10 years before the diagnosis of ED. Forty-two patients reported both age at STE and age at onset of ED (see Figure 5). The median time elapsed between STE and onset of the ED was 0 years [−3; 2], with some patients reporting that the STE happened over 10 years before the onset of the ED while others reported that the STE happened over 5 years after the onset of the ED.

## Discussion

The aim of this study was to assess the prevalence of various sexual traumatic events including sexual trauma in a clinical sample of children, adolescents, and adults with ED at time of assessment. Three hundred and twenty-nine patients diagnosed with an ED in a specialized ED unit were included.

Sixty-seven percent (95% CI [61%, 72%]) of the patients in our sample had experienced at least one TE, which is in line with the results in a study by Mitchell et al. (2012) where the vast majority of adult women and men with ED reported a history of at least one interpersonal trauma (Mitchell et al., 2012). In a Swedish study it was found that about 20% of the ED patients had experienced a TE, and 4.5% of the total sample had been exposed to at least one additional trauma (Backholm et al., 2013), which is a much lower frequency than our findings. There may be several explanations for these differences. All patients in our sample were included regardless of age, and a significant proportion of the patients were under the age of 18, which contrasts with the study by Backholm et al. (2013) where all participants under the age of 18 were excluded. Another contributing explanation may also be the methodological differences in the data collection of the trauma history. Our sample was assessed with an interview comprising more TE options than the Swedish study as we included bullying. Further, traumatic events may have been defined differently between the two studies.

Evaluation of the percentage of patients with ED that had experienced TE and putting this percentage into perspective is, however, challenging. When comparing the prevalence of the TE loss of a parent between our ED sample and the background population from the Danish registers (data from Statistics Denmark), we found that, in the 10–19 years age group, 2.4% of the background population had suffered the loss of a parent while 5% (95% CI [3%, 8%]) of the ED population had lost at least one parent. Thus, the prevalence of parental loss was somewhat higher in the ED population than in the general population. Thirty-two per cent (95% CI [27%, 38%]) of our ED population reported that they had experienced bullying. In a report investigating bullying among Danish students aged from 9 to 15 (in the year 2009–2010) (Dansk Center for Undervisningsmiljø, 2010) it was found that 16% had experienced bullying from other students. Although we cannot tell whether the bullying in our ED population was prior or posterior to the ED onset, the presence of bullying is clearly much higher in our ED population than seen in the report. We should stress that our patients report bullying at any time point in their lives while the report only included recent bullying. Still, it seems as if bullying is significantly more common in the ED population than in the background population.

Sixty-four of the females, corresponding to 19% (95% CI [15%, 24%]) of the patients, had been the victim of a sexual trauma or another sexual traumatic event. Of them, 13% had been the victim of a negative experience associated with sex, 57% had experienced sexual assault other than rape, and 30% had been the victim of severe forms of sexual assault. Comparing the number of sexual assaults in the study population to the general Danish population is challenging due to the dark figures as large numbers of offenses are unreported to the police (Socialstyrelsen: https://vidensportal.dk/temaer/seksuelle-overgreb/omfang; http://web.archive.org/web/20210124083808/https://vidensportal.dk/temaer/seksuelle-overgreb/omfang). However, a Danish report from 2008 may provide insight into sexual assault and violence in children and young adolescents (https://www.sdu.dk/da/sif/rapporter/2009/unges_trivsel_aar_2008; http://web.archive.org/web/20170320155123/Http://Www.Si-Folkesundhed.Dk/Upload/Unges_trivsel_2008_samlet_1.Pdf). The report was based on questionnaires delivered to 4093 students (approximately equal number of males and females) aged 14–17. The questionnaire included questions about sexual experiences, sexual experiences with adults, sexual assault, as well as other problematic experiences. About 22% of the females and 5% of the males reported that they had been subject to some type of unwanted sexual experience or sexual violence. When asked if they felt they had been sexually assaulted, 9% of the females and 1% of the males replied affirmatively. In contrast, 19% (95% CI [15%, 24%]) of this clinical ED population reported having been subjected to a STE. It is important to keep in mind that the setting for reporting the STE differs significantly between the two data sets. While the report used self-reported screening data, the reporting on STEs in our ED population was obtained during an interview with a psychologist at the end of a longer ED interview session in the hospital ward, i.e., the patients with ED were in a setting where they may have felt it was safe to reveal or discuss traumatic sexual experiences. There seems to be a higher prevalence of STEs in our study population of patients with an ED. However, this is only applicable for the females of this study as none of the male patients with an ED reported having experienced an STE. STEs may be more prevalent in the ED population and this could easily lead to the assumption that STE is a risk factor for the development of the ED. This would indeed be in accordance with the conclusion of a review on the relationship between ED and trauma, where it was concluded that childhood sexual abuse is a significant although non-specific risk factor for ED (Brewerton, 2007). However, as one in four of the patients who had experienced an STE in our ED sample reported having had ED symptoms before the time of the sexual trauma, the causality between the two is far from clear after all. As reported in *Results*, the prevalence of STE in adults with ED was 12 percentage points higher than in children with ED. Although this difference could be explained by time at risk, as adults have had a longer life and potentially thereby are at higher risk of being exposed to STE, the patients in the adult group reported having experienced STE at a young age. The prevalence of STE may vary depending on the severity and duration of the ED and it is therefore likely that the prevalence is higher in a subgroup of patients e.g., with severe and enduring ED.

There are both strengths and limitations to this cross-sectional study that should be mentioned. The study included a substantial number of patients assessed for an ED. Besides the sample size, another strength in the study is that all patients, regardless of age, gender and specific ED diagnoses, were included. As there is a limited number of studies of TE in children and adolescents with ED, this study does contribute with data in an area that is not well-documented but that is, nevertheless, highly relevant. However, some limitations should be mentioned. Although data relating to traumatic experiences were collected in a rigorous and uniform manner through interviews conducted by experienced clinical psychologists, this was carried out without the use of a recognized assessment instrument such as, e.g., Pennebaker and Susmans: Childhood Trauma Questionnaire (Pennebaker and Susman, 2013). The risk of recall bias when asked about traumatic experiences in the past may also be a considerable limitation for this study as well as for other studies of this topic. However, it is impossible to estimate the magnitude of this. The lack of a control group with a similar age and sex composition to our clinical sample poses a challenge when interpreting prevalence. Relevant references and publicly available data have been used to compensate for this absence. The age of the data set should be mentioned as a limitation. As the data set consists of clinical data subsequently released and approved for use in this research study there is a delay in publishing these results. As BED was not treated at the time of inclusion to the study, patients with BED could not be included.

## Conclusion

In conclusion, the majority of patients with ED reported that they had experienced at least one traumatic life event, and about one in five reported that they had been the subject of a negative sexual experience or sexual abuse. Although most of the patients with an ED had not experienced any sexual traumatic event, sexual trauma seems to be prevalent to an extent that makes it clinically relevant to address when assessing and treating patients with an ED. The causality between having an ED and having experienced a traumatic event of a sexual nature is, however, not clear as one in four experiencing a sexual trauma in our sample reported having had ED symptoms before the time of the traumatic sexual event. While the AN-R group reported a somewhat lower prevalence of STE than AN-BP, BN, and atypical AN, they reported the highest percentage of extreme sexual trauma of all diagnostic subgroups. This could be relevant to investigate in future studies.

Increased knowledge regarding traumatic life events and any possible causality between the ED and traumatic events are relevant objects for future research.

## Data Availability Statement

The datasets presented in this article are not readily available because of the severity of the topic. We will be happy to provide raw and anonymized data if requested but no micro-data can be provided. Requests to access the datasets should be directed to Gry Kjaersdam Telléus, gdkt@rn.dk.

## Author Contributions

GKT has participated in all parts of the study, design of the study, data collection, data management, statistical analysis, writing, and editing of the manuscript. ML participated in the writing of the manuscript. MR-D has participated in data management, statistical analysis, writing, and editing of the manuscript. All authors contributed to the article and approved the submitted version.

## Conflict of Interest

The authors declare that the research was conducted in the absence of any commercial or financial relationships that could be construed as a potential conflict of interest.

## Publisher's Note

All claims expressed in this article are solely those of the authors and do not necessarily represent those of their affiliated organizations, or those of the publisher, the editors and the reviewers. Any product that may be evaluated in this article, or claim that may be made by its manufacturer, is not guaranteed or endorsed by the publisher.
